# Alcohol Dependence in Rats Is Associated with Global Changes in Gene Expression in the Central Amygdala

**DOI:** 10.3390/brainsci11091149

**Published:** 2021-08-29

**Authors:** Brent R. Kisby, Sean P. Farris, Michelle M. McManus, Florence P. Varodayan, Marisa Roberto, R. Adron Harris, Igor Ponomarev

**Affiliations:** 1 Department of Pharmacology and Neuroscience, Texas Tech University Health Sciences Center, Lubbock, TX 79430, USA; brent.kisby@ttuhsc.edu (B.R.K.); michelle.mcmanus@ttuhsc.edu (M.M.M.); 2 Department of Neuroscience, University of Texas at Austin, Austin, TX 78715, USA; farrissp@pitt.edu (S.P.F.); harris@austin.utexas.edu (R.A.H.); 3 Department of Anesthesiology and Perioperative Medicine, University of Pittsburgh, Pittsburgh, PA 15261, USA; 4 Department of Biomedical Informatics, University of Pittsburgh, Pittsburgh, PA 15206, USA; 5 Department of Psychology, Binghamton University-SUNY, Binghamton, NY 13902, USA; fvaroday@binghamton.edu; 6 Department of Molecular Medicine, The Scripps Research Institute, La Jolla, CA 92037, USA; mroberto@scripps.edu; 7 Waggoner Center for Alcohol and Addiction Research, University of Texas at Austin, Austin, TX 78741, USA

**Keywords:** RNA-Seq, central nucleus of the amygdala (CeA), chronic intermittent alcohol vapor, extracellular matrix, alcohol use disorder (AUD), differentially expressed genes (DEGs)

## Abstract

Alcohol dependence is associated with adverse consequences of alcohol (ethanol) use and is evident in most severe cases of alcohol use disorder (AUD). The central nucleus of the amygdala (CeA) plays a critical role in the development of alcohol dependence and escalation of alcohol consumption in dependent subjects. Molecular mechanisms underlying the CeA-driven behavioral changes are not well understood. Here, we examined the effects of alcohol on global gene expression in the CeA using a chronic intermittent ethanol (CIE) vapor model in rats and RNA sequencing (RNA-Seq). The CIE procedure resulted in robust changes in CeA gene expression during intoxication, as the number of differentially expressed genes (DEGs) was significantly greater than those expected by chance. Over-representation analysis of cell types, functional groups and molecular pathways revealed biological categories potentially important for the development of alcohol dependence in our model. Genes specific for astrocytes, myelinating oligodendrocytes, and endothelial cells were over-represented in the DEG category, suggesting that these cell types were particularly affected by the CIE procedure. The majority of the over-represented functional groups and molecular pathways were directly related to the functions of glial and endothelial cells, including extracellular matrix (ECM) organization, myelination, and the regulation of innate immune response. A coordinated regulation of several ECM metalloproteinases (e.g., *Mmp2*; *Mmp14*), their substrates (e.g., multiple collagen genes and myelin basic protein; *Mbp*), and a metalloproteinase inhibitor, *Reck*, suggests a specific mechanism for ECM re-organization in response to chronic alcohol, which may modulate neuronal activity and result in behavioral changes, such as an escalation of alcohol drinking. Our results highlight the importance of glial and endothelial cells in the effects of chronic alcohol exposure on the CeA, and demonstrate further insight into the molecular mechanisms of alcohol dependence in rats. These molecular targets may be used in future studies to develop therapeutics to treat AUD.

## 1. Introduction

The current Diagnostic and Statistical Manual of Mental Disorders (DSM-5) integrates the two DSM–IV disorders, alcohol abuse and alcohol dependence, into a single disorder called alcohol use disorder (AUD) [1]. The severity of AUD is based on the number of criteria a person meets, with 6 or more out of 11 criteria indicating severe AUD [1]. Alcohol dependence is evident in most advanced AUD cases and is associated with adverse consequences of alcohol (ethanol) use, as well as indicators of alcohol tolerance, withdrawal, and uncontrolled drinking. Rodent models of alcohol dependence have been widely used to study the molecular and cellular mechanisms underlying the progression of AUD. The chronic intermittent ethanol (CIE) vapor inhalation method consistently produces physical dependence in mice and rats, as expressed by behavioral signs of withdrawal as well as increased alcohol drinking and anxiety-like behaviors [2,3,4,5,6,7]. The central nucleus of the amygdala (CeA) is implicated in the negative affective state of alcohol dependence and plays a key role in regulating stress-related behaviors and dependence-associated escalation of alcohol consumption [8,9,10,11,12,13,14,15,16,17,18]. As a result, alcohol-induced molecular and cellular changes in the CeA have been proposed to contribute to the pathophysiology of AUD [5,10,19]. Of note, alcohol-related physiological changes at a whole cell level and the role of different neuronal populations in the CeA have been studied extensively [5,9,10,20,21,22,23,24,25,26,27,28,29,30]. For example, one study showed that inactivation of a specific dependence-induced neuronal ensemble in the CeA reversed excessive alcohol drinking and somatic signs of alcohol dependence in rats [27]. However, the molecular changes underlying these cellular and behavioral responses to alcohol are not well understood.

Perturbation-induced gene expression serves as a sensitive measure of changes in cell functions, and numerous studies have used transcriptome profiling to investigate the mechanisms underlying brain plasticity and brain pathology [8,31,32,33,34,35,36,37,38,39,40,41,42]. Alcohol causes widespread changes in gene expression in the brain [31,39,42,43,44,45,46,47,48], some of which contribute to the development of AUD. Because AUD is a complex disease, with various brain circuits playing particular roles at different stages of AUD progression, it is important to determine alcohol-induced molecular changes across brain regions, AUD stages and animal models. To date, numerous alcohol-related gene expression studies have generated a wealth of transcriptomic data, providing insight into the molecular mechanisms of AUD [8,31,32,33,35,36,37,41,42,49,50,51,52,53,54,55]. In the present study, we complemented this valuable resource with transcriptomic profiling of the CeA in a rat model of alcohol dependence. We identified individual genes, biological functional groups and molecular pathways as mechanistic candidates for alcohol-induced behavioral changes. We used published cell type-specific molecular markers to define cellular identity of alcohol-regulated genes and to propose mechanistic roles for individual cell types in alcohol dependence. These cell type-specific molecular targets may be used in future studies to develop therapeutics to treat AUD.

## 2. Materials and Methods

### 2.1. Animals and Chronic Intermittent Ethanol (CIE) Exposure

All procedures were approved by The Scripps Research Institute (TSRI) Institutional Animal Care and Use Committee and were consistent with the National Institutes of Health Guide for the Care and Use of Laboratory Animals. Adult male Sprague Dawley rats (ordered at 200–250 g; 330–360 g at sacrifice) were obtained from Charles River Laboratories (Raleigh, NC) and randomly assigned into Ethanol (CIE) and Control groups (n = 6 per group). All rats were group housed throughout the study (n = 3 per cage), with *ad libitum* access to food and water. CIE rats received 5–7 weeks of daily ethanol vapor (14 h vapor/10 h air) with a target blood alcohol concentration (BAC) of 175–250 mg/dl, as previously described [5]. BACs were measured 1–2 times/week by tail-bleeding and upon sacrifice, and the mean BAC of the subset of rats used in this study was 188 ± 4 mg/dL. Rats from the Control group were treated similarly except with continuous air exposure. Animals were anesthetized with isoflurane and decapitated at the end of the last vapor exposure, and their brains dissected and split into two hemispheres for either gene expression analysis or electrophysiological recordings. Electrophysiological data were published previously [5]. Randomly chosen hemispheres were shipped on dry ice to the University of Texas at Austin for the gene expression analysis.

### 2.2. Gene Expression Using RNA-Seq

Brains were mounted in OCT and cryosectioned (300 µm coronal sections). The CeA was identified [56] and removed using a tissue puncher 1 mm in diameter (Stoelting, Wood Dale, IL, USA). CeA total RNA was isolated using the MagMAX™-96 Kit (Life Technologies, Carlsbad, CA, USA) and checked for quality control (all RIN values were >8.6). RNA library preparation and sequencing occurred locally (https://wikis.utexas.edu/display/GSAF). Illumina NextSeq of poly-A enriched total RNA sequencing was performed (PE 2 × 75, average of 40 million reads per sample). Individual sample libraries were mapped to *Rattus norvegicus* (Rnor_6.0; https://useast.ensembl.org/Rattus_norvegicus/Info/Index) reference genome using Burrows–Wheeler Aligner (BWA) [57]. Aligned sequencing reads were quantified using the python-based library HTSeq. Quantified expression data was analyzed for differential expression between treatment groups using the R Bioconductor package DESeq2 (v1.26) [58] within RStudio (v. 3.6.3), producing fold change, *p* values, and estimated false discovery rate (FDR).

### 2.3. Bioinformatics Analysis

To nominate candidate differentially expressed genes (DEGs) and biological groups we used two approaches, the first highlighting individual DEGs using a 5% FDR threshold and a convergent validity approach that combines nominal statistical significance and biological significance of bioinformatics analysis to control for Type 1 and Type 2 error rates. For the second approach, a list of genes differentially expressed between the two groups at a nominal *p* < 0.05 was subjected to bioinformatics analysis using two resources: (1) EnrichR (http://amp.pharm.mssm.edu/Enrichr, accessed on 1 July 2020), which identifies over-represented functional groups and molecular pathways using several well-curated databases including Gene Ontology (GO), KEGG and Wiki pathways, and (2) Ingenuity Pathway Analysis (IPA, www.ingenuity.com, accessed on 1 July 2020), a knowledgebase that identifies perturbation-related biological pathways and gene networks. In addition, a public database containing molecular markers of different brain cell types [59,60] was used to define cellular identity of DEGs. The criterion for a cell type-specific marker was at least 3-fold enrichment in a given cell type compared to a cell type with the second highest abundance. Molecular markers for astrocytes, neurons (general neuronal markers), oligodendrocyte progenitor cells (OPCs), myelinating oligodendrocytes, microglia and endothelial cells were used in the analysis. Over-representation *p*-values for each functional group, biological pathway, gene network and cell type were calculated using a hypergeometric test. The total number of DEGs, as well as numbers of up- and down-regulated DEGs, in specific cell types and functional groups were compared to chance using a Χ^2^ test with a Bonferroni correction. Finally, we searched for DEGs that are mechanistic candidates for cellular changes observed in our previous study using the same rat model [5]. Specifically, we focused on corticotropin releasing factor (CRF) and calcium channel systems. Because we obtained molecular and electrophysiological data from the same animals, we were able to correlate expression of DEGs with spontaneous GABA_A_-mediated inhibitory postsynaptic current (sIPSC) frequencies recorded from medial CeA neurons.

### 2.4. DEG Validation with qRT-PCR

Total RNA was reverse transcribed using Applied Biosystems High-Capacity cDNA Reverse Transcription Kit (Thermo Fisher Scientific Inc., Rockford, IL, USA) and then Taqman Fast Advanced Master Mix (Thermo Fisher Scientific, Rockford, IL, USA) were used to perform quantitative reverse transcription PCR (qRT-PCR). Applied Biosystems Taqman Gene Expression Assays included *Mmp14* (Rn01489226_g1), *Plp1* (Rn01410492_m1), *Gapdh* (Rn01775763_g1), and 18s (Hs99999901_s1). Reactions containing 5 ng of cDNA were performed in triplicate on the CFX384 Real-Time System (BioRad, Hercules, CA). Relative expression was determined using the 2^−ΔΔCt^ method and samples were normalized to the geometric mean of 18s and *Gapdh*. One statistical outlier from the Control group determined by the Grubbs test was removed and results were compared using a Student’s *t*-test with a threshold of *p* < 0.05 as statistical significance (Graph Pad 8.0.0).

## 3. Results

**Figure 1:**
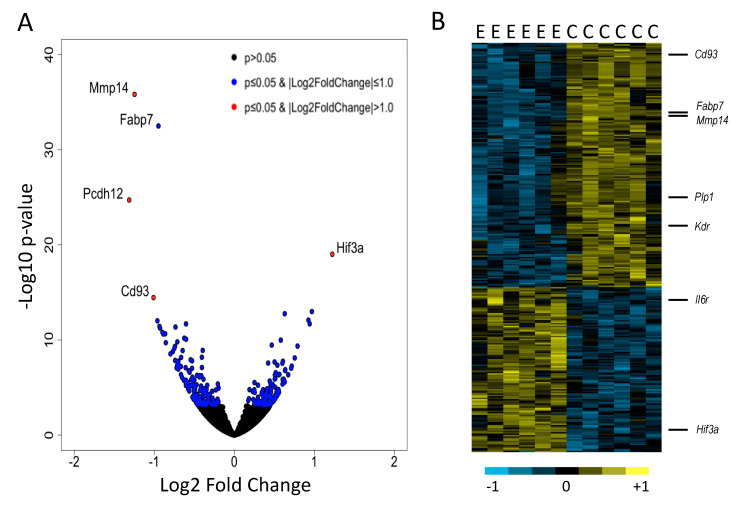
(**A**) Volcano plot showing differentially expressed genes (DEGs) (in color). Highlighted are the top statistically significant DEGs, up-regulated in the CIE group (on the right) and down-regulated in the CIE group (on the left). (**B**) Heat map of DEGs at 5% false discovery rate. Ethanol CIE group (E) and control air group (C). Representative cell and tissue type-specific genes are shown on the right.

**Figure 2:**
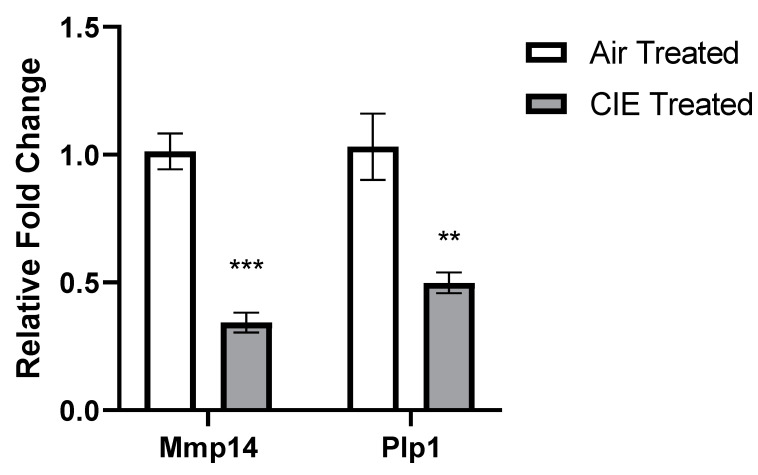
qRT-PCR validation of two cell type-specific DEGs, *Mmp14* (astrocytes) and *Plp1* (oligodendrocytes), differentially expressed at FDR < 5%. *** *p* < 0.001; ** *p* < 0.01 based on Student’s *t*-test.

The main objective of this analysis was to identify individual genes, functional groups and molecular pathways affected by chronic intermittent ethanol (CIE) in the CeA. Overall, 1837 genes were differentially expressed (DEGs) between the CIE and Control groups at a nominal *p* value of <0.05, with 985 DEG being up-regulated and 852 DEGs being down-regulated. Two hundred and eighty-five genes reached the statistical threshold of 5% FDR, with 115 DEGs being up-regulated and 170 DEGs being down-regulated (Figure 1, Supplemental Table S1). The total number of DEGs was significantly greater than those expected by chance (Χ^2^ *p* < 1.0 × 10^−7^), indicating marked effects of CIE on global CeA gene expression. Many top statistical DEGs were cell type- and tissue type-specific. For example, the top two statistical DEGs, matrix metallopeptidase 14 (*Mmp14*) and fatty acid binding protein 7 (*Fabp7*) are highly enriched in astrocytes. The C-type lectin transmembrane receptor, *Cd93* and the vascular endothelial growth factor receptor 2, *Kdr*, are markers of endothelial cells, whereas the proteolipid protein 1, *Plp1*, is a marker of oligodendrocytes. We validated RNA-Seq data of two cell type-specific genes using qRT-PCR (Figure 2). Astrocyte-specific *Mmp14* and oligodendrocyte-specific *Plp1* genes were shown to be down-regulated in the CIE group compared to control using both techniques. Future studies will validate prioritized DEGs at a protein and functional levels.

**Figure 3:**
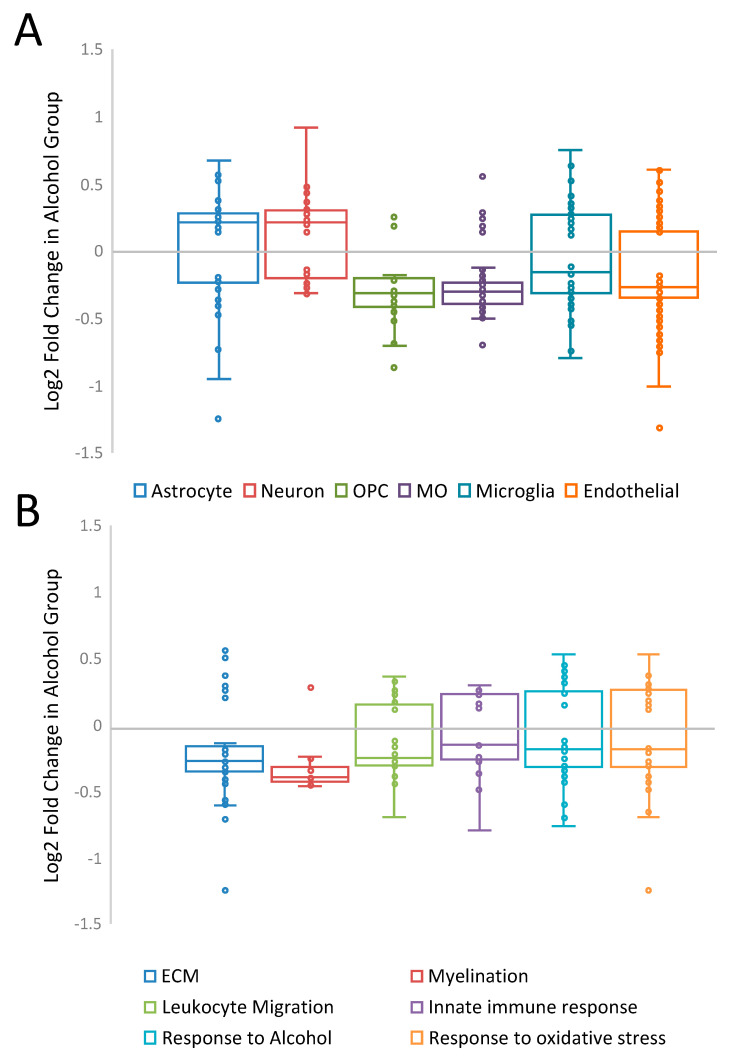
General directionality of CIE-induced gene expression changes within major CNS cell types (**A**) and selected over-represented functional groups (**B**). Individual DEGs are represented by small circles. Median expression of DEGs for each biological category is shown as a horizontal line within the 50% interquartile range. OPC: oligodendrocyte progenitor cells; MO: myelinating oligodendrocytes; ECM extracellular matrix.

Over-representation analysis of cell types, functional groups and molecular pathways revealed biological categories potentially important for the development of alcohol dependence in our model (Table 1, Supplemental Table S2). Genes specific for astrocytes, myelinating oligodendrocytes, and endothelial cells were over-represented in the DEG category, suggesting that these cell types were particularly affected by the CIE procedure. The majority of the over-represented functional groups and molecular pathways were directly related to the functions of glial and endothelial cells, including extracellular matrix (ECM), myelination, vasculogenesis, and regulation of innate immune response, pointing to the importance of non-neuronal cells in responses to chronic alcohol and the development of alcohol dependence. The majority of oligodendrocyte-specific and endothelial genes were down-regulated (all adjusted Χ^2^ *p* < 0.005), while astrocyte- and neuron-specific DEGs had a tendency to be more up-regulated (Figure 3A). The majority of DEGs from two highly over-represented functional groups, ECM and myelination, were also down-regulated (both adjusted Χ^2^ *p* < 0.005, Figure 3B). ECM organization was one of the top over-represented functional groups and included several metalloproteinases, including *Mmp2*, *Mmp14*, *Mmp15*, *Adam17*, *Adamts4*, which were all down-regulated in the CIE group; in contrast, *Adam8* and a metalloproteinase inhibitor, *Reck*, were up-regulated (Supplemental Table S1). Several known substrates of metalloproteinases were also regulated by alcohol, including several down-regulated collagen genes (*Col1a1*, *Col1a2*, *Col3a1*, *Col4a1*, *Col4a2*, *Col4a5*, *Col5a3*) and myelin basic protein (*Mbp*). One of the top statistically significant DEGs was interleukin 6 receptor (*Il6r*) (Figure 1B). This receptor is part of the pro-inflammatory cytokine IL-6 pathway that has been involved in the neuroimmune response to alcohol and may play a critical role in alcohol dependence [61,62,63].

**Figure 4:**
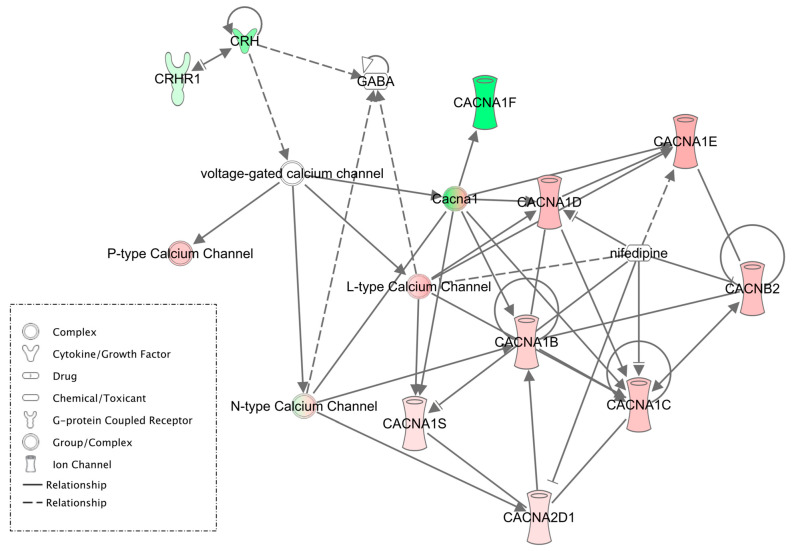
IPA-based molecular network showing literature-based relationships among calcium channels, CRF, and GABA. Corticotropin-releasing hormone (*Crh*) as well as L-type calcium channels, *Cacna1f* (Cav1.4) and *Cacna1d* (Cav1.3) were differentially expressed between CIE and control animals. Another CRF system-related DEG, *Crhbp* is not shown (see text for detail). Green color = down-regulation and red color = up-regulation in the CIE group, compared to control. These changes serve as molecular correlates of alcohol-induced cellular and behavioral effects mediated by CeA ([5]).

The present set of experiments was part of a larger study, with the electrophysiological, pharmacological and biochemical data published previously [5]. Of note, in this manuscript we will use the term corticotropin releasing factor (CRF) when referring the neurobiological actions of the peptide system and the equivalent term, corticotropin releasing hormone (CRH), as it relates to the gene symbol nomenclature. The Varodayan and colleagues paper highlights the importance of L-type calcium channels (LTCC) and the CRF system in mediating the effects of alcohol dependence on CeA gamma aminobutyric acid (GABA) neuron activity, and its role in the escalated alcohol intake in alcohol-dependent rats. To uncover molecular determinants of these neurobiological effects we searched for DEGs related to the LTCC and CRF systems. We found that two different types of LTCC and two CRH-related genes were changed in the CIE group, compared to control. Specifically, LTCC *Cacna1f* (Cav1.4) was down-regulated, whereas LTCC *Cacna1d* (Cav1.3) was up-regulated. In addition, *Crh* was down-regulated, whereas corticotropin releasing hormone binding protein, *Crhbp*, was up-regulated (Supplemental Table S1). An IPA-based gene network shows literature-based relationships between calcium channels and CRF and GABA systems (Figure 4). Correlational analysis of DEG expression with previously published sIPSC frequency values identified 220 statistically significant correlations (nominal *p* < 0.05) (Supplemental Table S1). In particular, *Cacna1f* (Cav1.4) was negatively correlated with sIPSC frequency (which reflects basal CeA GABA release), supporting its potential role in alcohol dependence.

## 4. Discussion

We identified numerous genes and functional groups regulated in the CeA of alcohol-dependent rats. Many of these genes are expressed in a cell type-specific manner and many of the functional groups represent known functions of specific brain cells, providing a more focused interpretation of the data. The CeA is a key brain region implicated in the regulation of escalated alcohol drinking in alcohol-dependent subjects [32,64,65,66,67,68]. It is the major output nucleus of the amygdala, and is connected to other parts of the extended amygdala, as well as other key brain regions involved in the regulation of alcohol effects. Therefore, transcriptional changes in CeA could significantly influence the activity of other brain regions [10,13]. The CIE vapor treatment produces a robust escalation of alcohol consumption [2,7,22,69], a hallmark of alcohol dependence, and we hypothesize that the identified molecular changes may be mechanistically linked to the CeA-mediated behavioral effects.

Our dataset is complementary to previous transcriptomic studies focusing on AUD models [8,22,31,32,35,36,37,42,52,68,69]. The previous work investigated various brain regions, including the CeA, using different alcohol paradigms, and mainly focused on time points corresponding to acute and protracted withdrawal from chronic alcohol (1 h to 3 weeks). For example, Repunte-Canonigo and colleagues [53] used a rat model of dependent alcohol self-administration to study transcriptional changes in the CeA and other brain regions at 3 weeks after the end of alcohol vapor exposure. The study focused on molecular networks of the glucocorticoid receptor, *Nr3c1*, and their role in alcohol dependence-induced drinking, and no cell type-specific analysis was performed. Compared to this and other studies, we investigated gene expression at the end of last alcohol vapor session, when the dependent animals were still intoxicated. We hypothesize that transcriptional changes at this time point reflect cellular adaptations to long-term alcohol exposure, some of which may contribute to behavioral phenotypes associated with alcohol dependence, such as behavioral tolerance, withdrawal severity, and escalated alcohol consumption. In our study, no behavioral measurements were obtained, and, therefore, no correlations with gene expression can be measured, a weakness that can be addressed in future experiments. Such an analysis may hint at which DEGs are causative to alcohol-related behaviors and which ones are simply compensatory to alcohol effects.

Our cell type-specific approach highlighted the importance of glial and endothelial cells in chronic alcohol effects on the CeA, as numbers of cell type-specific DEGs were greater than those expected by chance in astrocytes, oligodendrocytes and endothelial cells. Interestingly, the *Nr3c1* gene differentially expressed in the Repunte-Canonigo et al. study is enriched in these cell types [59], and, although it was not regulated in our study, this further highlights the importance of these cell types in alcohol dependence and provides some validation of our approach. Not surprisingly, biological functions typically associated with these cell types were also over-represented in the DEG list, including ECM organization, myelination, leukocyte migration, angiogenesis, vasculogenesis and a number of immune functions and molecular pathways. Myelin dysfunction has long been implicated in the effects of alcohol on the brain [70,71] and recent studies have also implicated ECM reorganization and neuroimmune processes in AUD-related conditions including alcohol dependence [35,64,72,73,74,75]. Microglia, the resident immune cells of the central nervous system, play an important role in brain normal processes and disease, including AUD. Recent reports showed that CIE treatment resulted in robust changes in gene expression in isolated microglia and astrocytes [76] and implicated these cell types in the regulation of alcohol consumption and development of alcohol dependence in mice [77,78,79]. Our data complement these findings by identifying specific molecular processes associated with glial functions in alcohol dependence in rats.

ECM organization was a top over-represented functional group in our study, with several different families of ECM genes being regulated, including matrix metalloproteinases (MMP), a disintegrin and metalloproteinases (ADAM), ADAM with thrombospondin motifs (ADAMTS), and collagen factors. Metalloproteinases are capable of digesting ECM macromolecules and non-ECM molecules, including some membrane proteins, growth factors, cytokines, collagen and myelin basic protein, all of which are determinants of the tissue microenvironment [72,80,81]. In the brain, metalloproteinases are critical for tissue formation, neuronal network remodeling, and blood–brain barrier integrity [72,82,83]. MMPs are Zn^+2^ dependent endoproteinase that are important for the cleavage of extracellular proteins and inactivation of certain chemokines and cytokines (i.e., cleavage of TNF-α, glycoproteins, and collagen) [84,85,86]. MMPs have been linked to several biological functions within the brain such as synaptic plasticity [65,72,87], upregulation in gliomas [88], chronic inflammatory diseases [89], AUD [72,87], and neuron/CNS repair mechanisms [83]. Similar to MMPs, ADAMs are membrane anchored enzymes that are regulated by Zn^+2^ and play a similar role to MMPs. Several genes from this family, including *Adam8* and *Adam17* were differentially expressed between the groups. A recent study proposed a role for ADAM8 in cell adhesion during neurodegeneration [90]. There is currently limited information on the role of *Adam8* as it relates to alcohol dependence. A quick literature search revealed a regulation of this gene in genetic mouse models of high alcohol consumption [35]. Regarding *Adam17*, a study by Bell and colleagues showed that there was a five-fold lower expression of *Adam17* in the nucleus accumbens of alcohol preferring (P) rats [48]. Interestingly, in a study of post-mortem brain samples in individuals with Schizophrenia and bipolar disorder, the levels of TNF-α were negatively correlated with those of *Adam17* [91], suggesting anti-inflammatory properties for this gene. Another ECM gene, reversion-inducing, cysteine-rich protein with Kazal motifs (*Reck*) was upregulated by alcohol. RECK is a glycosylphosphatidylinositol-linked glycoprotein, which inhibits MMP-2, MMP-9, and MT1-MMP (MMP-14) [80,92,93]. There is limited information on the role of RECK in AUD, with the exception that it was differentially expressed in the postmortem brains of AUD subjects comparted to control [51]. A report by Wang and colleagues implicated RECK in the protection of tissue integrity and promotion of functional recovery in the brain after cerebral ischemia [94], suggesting a possible compensatory role of *Reck* upregulation in response to alcohol-induced tissue damage. Gene expression studies in alcohol mouse models reported ECM as an over-represented functional group. For example, our recent study showed that a decitabine-induced decrease in voluntary alcohol consumption in non-dependent C57BL/6J male mice was associated with changes in several ECM genes in the ventral tegmental area [75]. Some of the genes (e.g., *Kdr*, *Adam17*) overlapped with our current study, while many others were different, suggesting that alcohol-related changes in ECM genes are, at least in part, specific to species, alcohol model, or brain region.

Collagens, that can serve as MMP substrate, are ubiquitous proteins that constitute the main structural element of the ECM, and their main function is participation in cell-to-cell adhesion. We identified several differentially expressed collagen-producing genes including *Col4a1*, *Col4a2*, *Col1a1*, *Col3a1*, *Col4A5*, *Col15A1*, *Col5a3*, *Col1a2*, *Col6a1*, *Col19a1*, and *Col22a1*. It has been shown that *Col4a1* mutations may lead to gross morphological changes to mouse brains as well as neurological inflammation and cortical hemorrhage [95,96]. *Col3a1* and *Col1a1* are the most abundantly expressed genes in the ECM. One study by Mouton and colleagues looked at the effects of *Col3a1* and *Col1a1* in the ECM of cardiac cells and showed that alcohol decreased the expression ratio of *Col3a1* and *Col1a1* [97]. This research group also showed that *Lox* was attenuated after alcohol administration in cardiac ECM [86,97]. Additionally, *Lox* is important for the cross-linking of collagen fibers [86,97].

A coordinated regulation of several ECM metalloproteinases including *Mmp2* and *Mmp14*, their substrates (e.g., several collagen genes and myelin basic protein, *Mbp*), and a metalloproteinase inhibitor, *Reck*, suggests a specific mechanism for ECM re-organization in response to chronic alcohol, which may modulate neuronal activity and result in behavioral changes, such as an escalation of alcohol drinking. This mechanism may link alcohol-induced demyelination, changes in endothelial cell functions and blood-brain barrier integrity, leukocyte migration and other immune responses to the action of several ECM metalloproteinases on their substrates. The majority of metalloproteinases in our study were down-regulated by alcohol. It is important to note that the direction of changes at an mRNA level does not necessarily imply the same direction at the protein or functional levels, as many mRNA changes indicate a compensatory response of a cell to a loss or gain of function [98,99,100]. mRNA changes simply implicate a biological process or function, and additional experiments at a functional level are necessary to define the exact mechanism.

It has been shown that CRF (*Crh*) and its primary receptor subtype 1, CRF1 (*Crhr1*) and more recently the binding protein, *Crhbp*, are implicated in several alcohol-related behaviors including, but not limited to, binge [101,102] and chronic alcohol exposure [103]. We have shown that alcohol dependence recruits the CRF system and alcohol consumption is strongly driven by the CRF1 receptors [4]. Our previous study highlighted the functional role of CeA CRF1 and L-type calcium channel signaling in the development of alcohol dependence [5]. Specifically, acute alcohol increased CeA neuronal activity in naive rats by engaging LTCCs, and intra-CeA LTCC blockade reduced alcohol intake in nondependent rats. Alcohol dependence disrupted this LTCC-based mechanism and revealed the importance of the CRF1 pathway in driving escalated alcohol drinking in dependent animals. Here, we found that *Crh*, *Crhbp* and 2 LTCC genes (*Cacna1f* and *Cacna1d*) were differentially expressed between the CIE and control groups, supporting their role in the mechanisms observed at the cellular and behavioral levels. Although the directionality of transcriptional regulation does not imply a gain or reduction of function, these four genes are primary molecular candidates for alcohol-induced CeA-mediated behavioral effects. It is currently not clearly understood how *Crhbp* interacts with CRF and CRF1 receptors. However, there is some evidence of alcohol effecting *Crhbp* in the VTA [104,105], PFC [106], and CeA [105]. For example, Haass–Koffler and colleagues showed that a selective reduction of *Crhbp* expression in the CeA decreases ethanol consumption in ethanol-dependent rats, a result consistent with an up-regulation of this gene in our ethanol-dependent animals, which are expected to drink more ethanol after the CIE treatment [105].

## 5. Conclusions

In summary, we propose a critical role for non-neuronal cells and cellular functions in the effects of chronic alcohol on the brain and the development of alcohol dependence. The specific role of metalloproteinases and other ECM molecules in the development of alcohol dependence remains unclear and warrants further investigation. The glial- and endothelial-related changes may contribute to changes in neuronal activity, which ultimately leads to the escalated alcohol intake in alcohol-dependent subjects. The current study nominates potential targets for developing therapeutics to treat AUD.

## Figures and Tables

**Table 1:** Over-represented cell types and representative biological functional groups and molecular pathways. For a full list of over-represented functional groups and pathways, see Supplemental Table S2.

Biological Category		# of Genes	*p* Value
Cell type			
	Myelinating Oligodendrocyte	60	9.20 × 10^−12^
	Endothelial Cells	100	5.40 × 10^−7^
	Astrocyte	46	3.00 × 10^−12^
Functional Group			
	Extracellular Matrix (ECM) organization	59	1.57 × 10^−6^
	Ensheathment of neurons	18	1.94 × 10^−5^
	Brain development	33	9.51 × 10^−5^
	Myelination	16	1.09 × 10^−4^
	Leukocyte migration	36	2.82 × 10^−4^
	Regulation of cell adhesion	47	5.28 × 10^−4^
	Regulation of cytokine production	62	5.60 × 10^−4^
	Response to alcohol	40	6.43 × 10^−4^
	Vasculogenesis	14	2.55 × 10^−3^
	Response to oxidative stress	39	2.83 × 10^−3^
	Regulation of innate immune response	34	5.41 × 10^−3^
	Regulation of blood vessel size	11	2.11 × 10^−2^
Molecular Pathway			
	NF-kappa B signaling pathway	16	1.09 × 10^−2^
	IL-6 signaling pathway	16	2.90 × 10^−2^
	IL-1 signaling pathway	8	3.01 × 10^−2^

## Data Availability

Raw RNA-seq data has been deposited into NCBI GEO Accession number GSE159136.

## Supplementary Materials

The following are available online at https://www.mdpi.com/article/10.3390/brainsci11091149/s1, Table S1: title: “A list of DEGs”, Table S2: title: “A list of over-represented biological functional groups”.
